# Electrochemical recycling of polymeric materials

**DOI:** 10.1039/d4sc01754d

**Published:** 2024-05-21

**Authors:** Weizhe Zhang, Lars Killian, Arnaud Thevenon

**Affiliations:** a Organic Chemistry and Catalysis, Institute for Sustainable and Circular Chemistry, Faculty of Science, Utrecht University Universiteitsweg 99 Utrecht The Netherlands a.a.thevenon-kozub@uu.nl

## Abstract

Polymeric materials play a pivotal role in our modern world, offering a diverse range of applications. However, they have been designed with end-properties in mind over recyclability, leading to a crisis in their waste management. The recent emergence of electrochemical recycling methodologies for polymeric materials provides new perspectives on closing their life cycle, and to a larger extent, the plastic loop by transforming plastic waste into monomers, building blocks, or new polymers. In this context, we summarize electrochemical strategies developed for the recovery of building blocks, the functionalization of polymer chains as well as paired electrolysis and discuss how they can make an impact on plastic recycling, especially compared to traditional thermochemical approaches. Additionally, we explore potential directions that could revolutionize research in electrochemical plastic recycling, addressing associated challenges.

## Abstract 2

Electrochemistry shows promising new avenues in the recycling of polymeric materials. This work reviews electrochemical depolymerization, post-polymerization functionalization, and paired catalysis, highlighting future challenges and opportunities.

## Graphical abstract

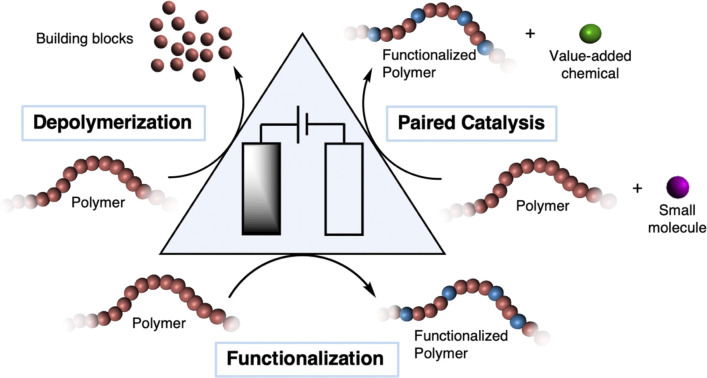

## 1 Introduction

Plastics are ubiquitous and essential to modern society. They are relatively inexpensive to manufacture and display many notable advantages over other materials such as light weight and processability, as well as tunable thermal, physical, and mechanical properties. By combining polymeric materials with diverse additives, compatibilizers, among other components, the material properties are tailored to make countless plastic products in industrial sectors ranging from packaging, agriculture, construction and automotive, to medicine and household. Since the beginning of the plastic era, over 8 billion tons of plastics have been produced. Most of these have been designed with specific end-user properties in mind, as opposed to their end-of-life management. Consequently, more than 60% of the plastic produced has ended up in landfills or is lost in the environment. This represents both a waste of potential resources as well as an ecological disaster.^1^

In Europe, approximately 65 Mt of plastic waste is collected every year.^2^ Half of it is shipped to non-EU countries and the other half is used for energy recovery (21%), and landfilled (13%), while only 16% is recycled. One of the reasons for this low percentage in recycling is that waste streams consist of mixtures of various plastics of unknown compositions and are potentially contaminated by organic (*e.g.*, food remains) or inorganic (*e.g.*, lead in polyvinylchloride (PVC)) fractions.^3^ Other reasons include the limited recycling technology, improper disposal procedures and lack of incentive policies. There are currently four types of strategies implemented for plastic recycling: reusing (primary), mechanical (secondary), chemical (tertiary) and energy recovery.^4^ Primary reusing strategies require uncontaminated and pristine-like quality feeds of single-use waste plastics representing a very small portion of the plastic waste stream.^5^ Mechanical recycling can give a second life to plastic waste by reprocessing it. However, recycled plastics typically show inferior mechanical/thermal properties compared to pristine feeds, due to the deterioration of the polymer backbone (*i.e.*, lower molecular weight from chain scission) and are therefore limited to few recycling cycles.^6^ Energy recovery, the process of burning plastic waste to produce energy in the form of heat, electricity, or fuel, has the advantage that it works effectively for any complicated mixtures of plastics, while it is at the cost of releasing greenhouse gases and/or toxic byproducts.^7^ Moreoever, current technologies are not able to recover incineration products, such as monomers or chemical building blocks, for reuse.

Chemical recycling offers an exciting opportunity to recover valuable products from polymeric materials, such as monomers, chemical building blocks, or new polymers from single plastic waste streams that are not compatible or cannot be recycled (any more) by the primary or secondary strategies.^5,8,9^ In chemical recycling, polymers can be classified into two categories: polymers with chemically cleavable units (*e.g.*, polyester, polyamide, polycarbonate, polyurethane) and chemically inert polymers (*e.g.*, polyolefins, PVC, polystyrene). In contrast to promising examples of industrial processes for the depolymerization of polyesters (*e.g.*, methanolysis process from Eastman, Volcat process from IBM, or catalytic PET process from Ioniqa),^10^ no industrial process exists for chemically inert polymers despite representing the biggest volume of plastic waste generated yearly.^1^ In other words, chemical recycling is still not commonly implemented on an industrial scale as it is energy-intensive, notably due to the various chemical treatments involved throughout the recycling process (*e.g.*, chemical reactions, extractions, and purifications).^5^ The challenges in implementing chemical recycling are motivating the search for novel mild processes for the catalytic conversion of polymers directly to monomers, building blocks and new polymers.

**Fig. 1:**
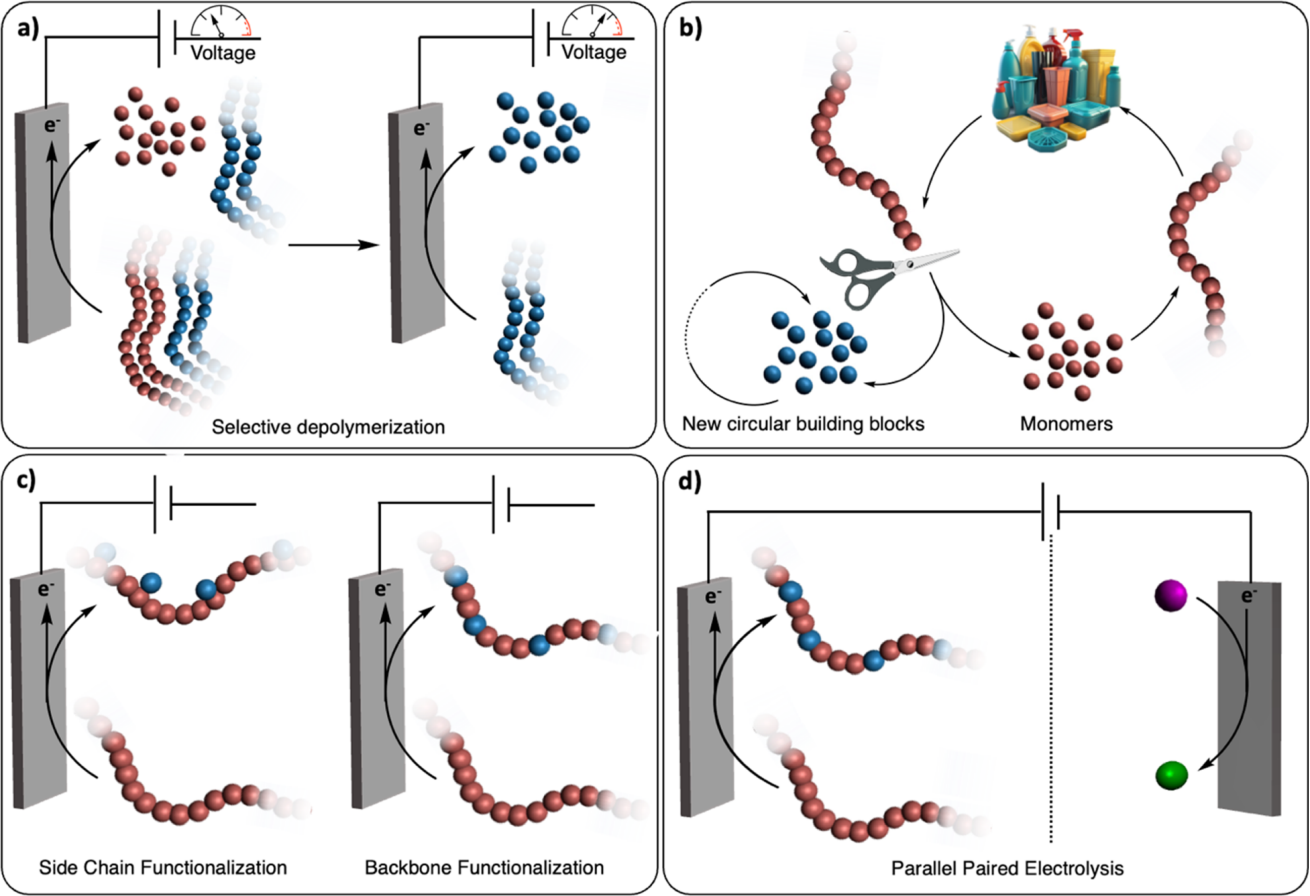
Electrochemical recycling of polymeric materials. (a) Electrochemical depolymerization of multilayered polymeric materials. Selective depolymerization of the first polymer component (red polymer) is achieved at a given electrochemical potential. The second polymer component (blue polymer) is then depolymerized at a higher potential. (b) General scheme for closed-loop polymer recycling. Polymers are either selectively depolymerized into monomers (red spheres) or other building blocks (blue spheres). In the former case, monomers can be repolymerized back to (the same) polymer and in the latter case, the building blocks can be used for other applications. (c) Post-polymerization functionalization. Functional groups are electrochemically added to the side chain or the backbone of the polymer. (d) Parallel paired electrolysis. The electrochemical oxidative functionalization of the polymer is performed simultaneously with the reduction of a small molecule (purple sphere) to another value-added product (green sphere).

The renaissance of (organic-)electrochemistry since the 2000s is contributing to fantastic breakthroughs in the development of novel methodologies to functionalize small molecules with outstanding precision (*i.e.*, chemo/regioselectivity) under mild conditions.^11^ We envision that these recent advancements will greatly inspire the emerging field of electrochemical recycling of polymeric materials and, to a greater extent, of plastics, to push the limits of traditional chemical recycling techniques (Fig. 1). For instance, electrochemistry offers direct control over the potential energy of electrons *via* applied voltages. This real-time tuning of catalyst activity and selectivity could be particularly advantageous for selectively recycling multilayered polymeric materials or even dealing with mixed plastic waste (Fig. 1a). Since electrochemistry uses traceless reagents (electrons) derived increasingly from renewable sources, it circumvents the use of potentially harmful and stoichiometric chemical reductants and oxidants which generates additional waste.^12^ Moreover, it allows for separated half-reactions, providing more opportunities than conventional thermochemical processes, for example through paired catalysis where two value-added products would be generated from a single (electro-)chemical reaction (Fig. 1d).^13^

Several recent reviews have examined the field of electrochemical recycling of polymeric materials, notably the review by Petersen *et al.*^14^ which offers a comprehensive review, principally focusing on biopolymers, until 2021.^12,15,16^ Given the rapid progress in electrochemically-driven recycling of synthetic polymers in recent years, we offer perspectives on the future trajectory of this field, incorporating discussions on the latest achievements. By contextualizing other studies on thermochemical and electrochemical functionalization of small molecules as well as functionalization/depolymerization of synthetic and biopolymers, we aim to provide the community with a source of inspiration for the development of new approaches. Depolymerization into building blocks, post-polymerization functionalization, and paired electrocatalysis are discussed based on the distinct requirements for their use in polymer and plastic recycling. These approaches cater to diverse needs, including complete cleavage into segments, repurposing plastic waste, and construction of a comprehensive electrochemical device (Fig. 1). Beyond conceptualizing and classifying these ideas of electrochemical recycling of polymers, we also discuss the challenges linked with the development of such novel technologies. From there, we speculate on new research directions that could spur the advancement of (electro-)chemical recycling technologies of polymeric materials and plastics.

## 2 Electrochemical recovery of building blocks

Chemical recycling to building blocks (CRBB) refers to approaches in which polymers are cleaved into small molecules such as their monomers (Fig. 1b). CRBB is an appealing and promising recycling strategy as valuable chemicals can be obtained from end-of-life polymers and plastics. Moreover, if the building blocks are recycled back to monomers, the original starting materials can be recovered, purified, and subsequently repolymerized into new polymers with identical properties to their virgin counterparts. This closed-loop approach ideally creates a circular polymer economy, wherein polymeric materials are continuously recycled and reused, reducing the need for new raw materials.^9^ For polymer backbones containing chemically cleavable bonds (*e.g.*, C–O bonds such as in esters and acetals), CRBB is particularly effective.

Herein, we will first discuss the recent progress in the development of electrochemical methods to cleave C–O bonds in the backbone of polymers for polyethylene terephthalate (PET), polyoxymethylene and biopolymers. We will then briefly present an example of S–S bond scission before discussing the more challenging scission of C–C bonds in polyolefins. To the best of our knowledge, electrochemical CRBB methods have not been developed for breaking C–N bonds to date; we therefore provide our perspective on new potential research directions based on our previous discussion on electrochemical C–O and C–C bond cleavage.

### 2.1. Electrochemical C–O bond cleavage

**Fig. 2:**
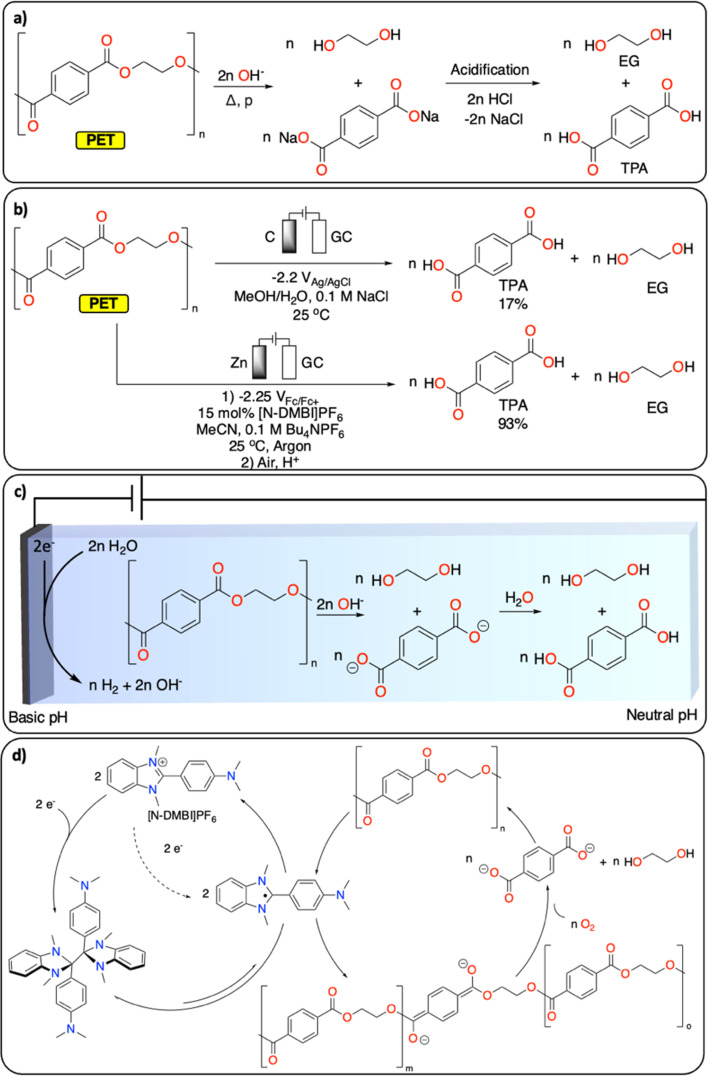
Depolymerization of PET to TPA and EG. (a) Hydrolysis under basic conditions; (b) electrochemical approaches reported;^20,21^ (c) electrochemically generated pH gradient;^21^ (d) redox-mediated reductive depolymerization.^20^

Polyesters and polycarbonates represent a major class of polymeric materials with versatile physical properties making them ubiquitous to a large range of applications from construction materials to medical applications.^17^ Among the polyester family, PET is the third most produced and discarded plastic worldwide.^1^ It is a semi-crystalline polymer and its thermochemical depolymerization has been widely studied.^18^ Thermochemical CRBB approaches are based on solvolysis methods (hydrolysis, glycolysis, alcoholysis), which enable the recovery of ethylene glycol (EG) and terephthalic acid (TPA), the two monomers of PET (Fig. 2a). Solvolysis methods require high temperature/pressure as well as extreme pH conditions to allow for high conversion.^18^ Furthermore, they can generate a substantial amount of (inorganic) waste during the neutralization step required, for instance, if the solvolysis is performed under basic conditions. Despite these limitations, solvolysis is used at large scale by several companies (*e.g.*, Eastman, IBM, Ioniqa, DuPont, Shell Polyester, Zimmer, and Goodyear).^10,19^

Electrochemically induced depolymerization is a great potential alternative that enables the recovery of EG and TPA under mild conditions (Fig. 2b). For instance, Luca *et al.* developed an electrochemical depolymerization strategy for PET that avoids the use of highly corrosive caustic solutions (Fig. 2c).^21^ By generating a local basic environment in a water/MeOH-based electrolyte, they were able to recover up to 17% of TPA within one hour at room temperature. These encouraging results motivate further development of electrochemical solvolysis methodologies. However, electrochemistry allows to go beyond simple solvolysis, notably by reductive catalytic depolymerization reactions, which are not possible using traditional thermochemical approaches. For instance, Luca *et al.* also demonstrated the electrochemical depolymerization of insoluble semi-crystalline PET particles using benzimidazolium cations ([*N*-DMBi]PF_6_]) as redox mediator to generate ester anion radicals, leading to almost quantitative depolymerization of PET into TPA and EG upon exposure of the reaction mixture to air after electrocatalysis (Fig. 2d).^20^

Once the depolymerization is completed, the recovery of TPA and EG is essential for subsequential repolymerization and chemical conversions. In contrast to the straightforward isolation of TPA (*via* precipitation from aqueous solution by changing the pH), the recovery of EG from aqueous solutions is more challenging. EG's high boiling point (197 °C) and excellent water solubility make the distillation energy intensive. Alternatively, electrochemical methods enable the direct transformation of EG into various value-added products. For instance, EG can selectively be oxidized to formate^22^ or glycolic acid (GA)^23^ (with the concomitant hydrogen evolution reaction), making the overall PET CRBB process more cost-effective (*cf.* part 3).^22^

More generally, polyesters and polycarbonates are characterized by a large electrochemical window making them attractive as electrolytes for battery and electrosynthesis applications, yet this same characteristic may restrict their potential for electrochemical depolymerization reactions.^24^ Consequently, other examples of electrochemical recycling of polyesters and polycarbonates beside PET remain scarce. Promisingly, Voloshchuk *et al.* demonstrated that bisphenol A can be recovered from polycarbonate *via* electrochemical reductive depolymerization in DMF, in the presence of Bu_4_NClO_4_, on a platinum electrode.^25^ Separately, Mindemark *et al.* reported that these polymers can decompose under high oxidative and reductive potentials on carbon and lithium electrodes, resulting in a mixture of oligomers, hydrocarbons (*e.g.*, C_3_H_8_), CO_2_ and CO.^26,27^ We envision that further development of redox mediators and efficient electrocatalysts could broaden the scope of reductive/oxidative depolymerization. This development has a great potential to enhance control over selectivity toward monomers or other building blocks and lower their onset potentials.

**Fig. 3:**
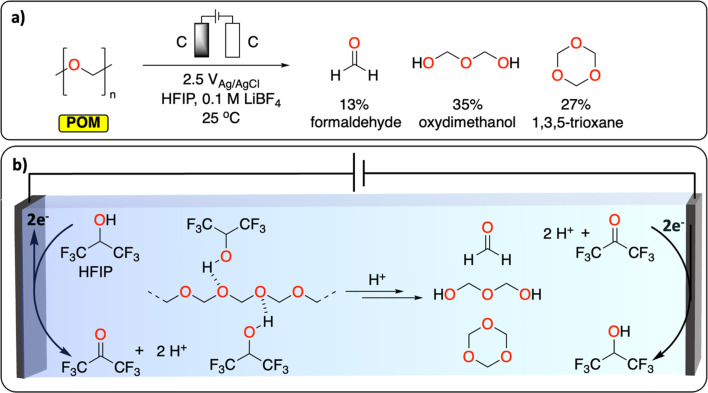
Electrochemical depolymerization of POM. (a) Reaction conditions reported to generate formaldehyde, formic acid and 1,3,5-trioxane *via* electrochemical depolymerization of POM. (b) The dual role of HFIP in breaking the crystallinity of POM and in generating H^+^ during its oxidation to trigger POM depolymerization.^28^

Polyether represents the last class of polymers containing C–O groups. The depolymerization of polyoxymethylene (POM) is a promising example of CRBB performed on highly crystalline polymers (Fig. 3). A total yield of over 50% consisting of various building blocks (mostly formaldehyde, oxydimethanol and 1,3,5-trioxane) could be recovered at ambient temperature, after 2.5 h in an undivided cell using graphite working and counter electrodes. The authors further highlight the dual role of the solvent (hexafluoropropanol, HFIP) in breaking down some of the crystalline parts of the polymer to expose more of the acetal linkages, and in liberating protons during its anodic oxidation to trigger POM depolymerization. This work demonstrates a new chemical tool for recycling insoluble and highly crystalline polymers under mild conditions; something that is especially challenging using thermochemical approaches.^28^

**Fig. 4:**
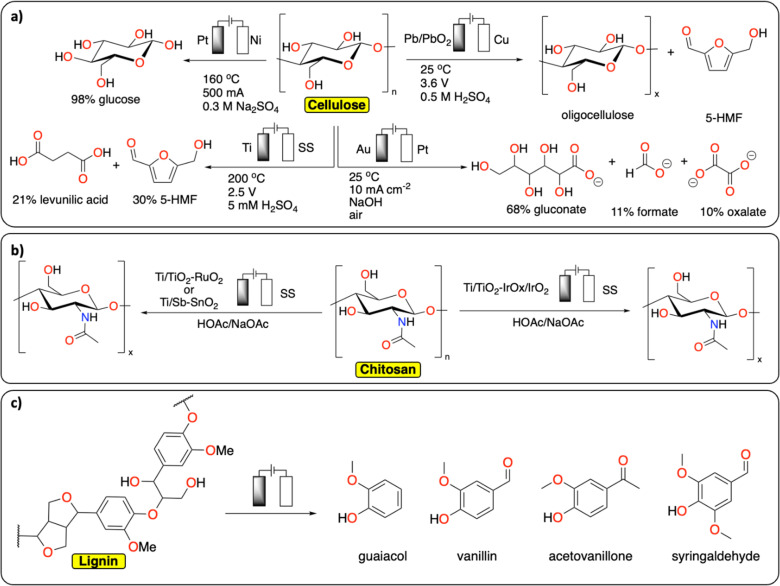
Electrochemical depolymerization of biopolymers. (a) Cellulose;^32–34,36^ (b) chitosan;^39,40^ (c) lignin and the corresponding products obtained. For lignin, only a general scheme is provided, we refer the readers to these reviews for a more detailed description of the existing methodologies.^43–49^

In comparison with synthetic polymers, the electrochemical depolymerization of biopolymers to recover building blocks (*e.g.*, glucose, furfural, 5-HMF, levulinic acid, monomers) is relatively far in development, with many studies published over recent years (Fig. 4).^29–31^ Most electrochemical depolymerization methods essentially follow traditional thermochemical approaches, with the added benefit of low reaction temperatures and pressures.^14^ The most common biopolymers studied are cellulose, lignin and chitin. Cellulose and chitin are both connected *via* β(1 → 4) acetal linkages, but differ in the structure of the monomeric sugars. While cellulose and chitin are semi-crystalline polymers, lignin, due to the heterogeneity of its composition, is amorphous with more variation in linkages and different monomers.

A number of electrochemical approaches have been developed to selectively cleave the β(1 → 4) linkages of cellulose to isolate glucose as a final product (Fig. 4a). One of the early reports details the electrochemical generation of an acidic solution in the anode chamber to hydrolyze cellulose at high temperatures in an electrochemical batch reactor.^32^ A maximum yield of 98% glucose could be obtained by applying 500 mA current on a 2.5 cm square Pt anode, at 160 °C. Besides glucose, 30% 5-HMF and 21% levulinic acid were recovered from cellulose in a different study, using a batch reactor with 5 mM H_2_SO_4_ electrolyte and a cell voltage of 2.5 V, at 200 °C.^33^ Several other approaches were investigated at room temperature. In these strategies, radicals formed at the anode facilitate the depolymerization reactions. Li *et al.* reported on a Pb/PbO_2_ anode that generates hydroxyl radicals in a sulfuric acid solution to depolymerize cotton into a mixture of oligomeric sugars.^34^ It is proposed the hydroxyl radicals formed at the anode oxidize the cellulose substrate. Similarly, Li *et al.* used Fenton-type chemistry to depolymerize cellulose using graphite/PTFE electrodes modified by 2-ethylanthraquinone.^35^ The total yield of soluble sugars and other products reached a maximum of 15.8%. In a different approach using alkaline conditions in conjunction with a gold-based electrode, Zhao *et al.* were able to obtain gluconate with a yield of 67.8%, together with lower yields of formate and oxalate.^36^ The ability to control the concentration of acidic species or radicals at the electrode surface has been further discussed in a publication by the group of Koper.^37^ In this study, the electrochemical hydrolysis and decomposition of cellobiose – a dimer of glucose – has been compared to the equivalent thermochemical reactions. In contrast to thermochemical methods, electrochemical conversion of cellulose benefits from milder reaction conditions and better control of radical-assisted oxidation.

Another common biopolymer is chitosan, which constitutes partially deacetylated chitin (Fig. 4b). As with cellulose, most electrochemical methods are aimed at partial depolymerization by selectively targeting the linkages between the monomeric glucosamine units. One of the first reports on chitosan depolymerization details the use of a Ti/TiO_2_–RuO_2_ anode in an aqueous acetic acid/sodium acetate solution.^38^ Using this method, the authors successfully decrease the degree of polymerization without notable deacetylation. In a similar study, a Ti/Sb–SnO_2_ electrode was used with increased performance compared to the study mentioned before.^39^ Other electrode materials have also been used successfully to decrease the degree of polymerization in chitosan, such as an iridium based electrode, Ti/TiO_2_–IrO_*x*_/IrO_2_, used by Hu *et al.*^40^ Another interesting finding from Zeng *et al.* could inspire new ideas in the field of plastic electrochemical recycling.^41^ They reported an approach consisting of inducing partial depolymerization of chitosan directly on solid particles. They found that by using a pulsed electric field treatment of 25 kV cm^−1^, the crystalline phase of the polymer was significantly damaged inducing the degradation of high molecular weight chitosan. Relating back to plastic recycling, the development of pulsed-electrochemical methods could be of high interest for breaking the crystalline phase of polymers.

Lignin constitutes a key component of lignocellulosic biomass and its valorization has gathered substantial interest over recent years.^42^ In extension to this, it is also one of the most studied polymers in terms of electrochemical reactivity (Fig. 4c). Due to the heterogeneity of its composition, many different products can be formed from its depolymerization and subsequent reactions. These different products consist of a wide range of low molecular weight compounds, mostly different phenolics. There are both oxidative and reductive methods known for the electrochemical conversion of lignin, which mostly follow thermochemical approaches.^14^ The current state of the art and limitations in electrochemical lignin conversion have been extensively reviewed in recent publications.^43–49^ Deng *et al.* notably mention four main challenges that need to be overcome before electrochemical processes for lignin conversion can become viable.^44^ These are unfavourable side-reactions (*e.g.*, water splitting), low selectivity, overoxidation (*e.g.*, decarboxylation and generation of CO_2_) and limited electrolyte options. Besides lignin, the electrochemical conversion of lignin model compounds has also been studied extensively in recent years.^50^ Because of the wide variety of products that are obtained in these reactions, the main developments in this field concern ways of steering selectivity and isolating products.

### 2.2. Electrochemical S–S bond cleavage

**Fig. 5:**
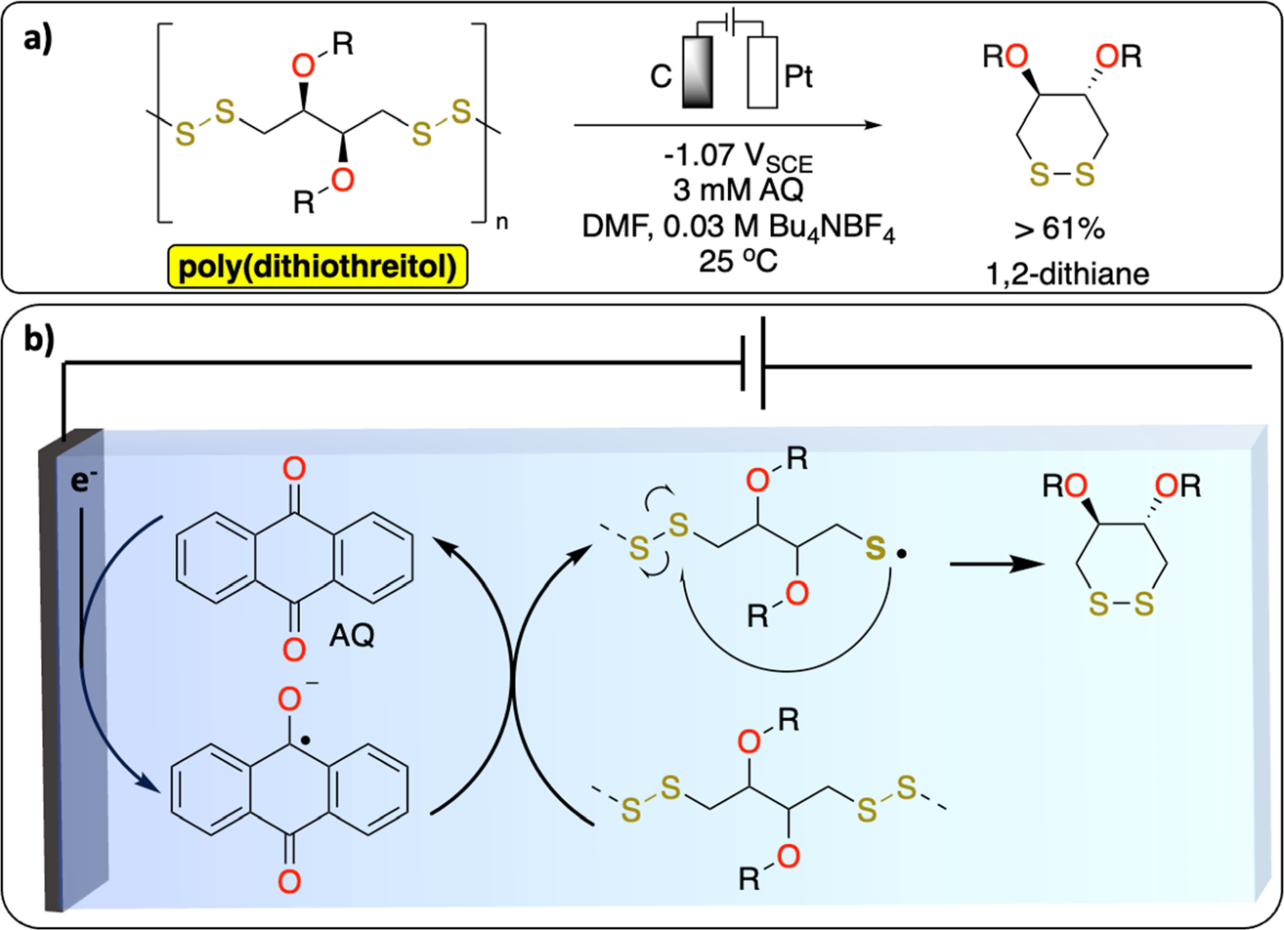
Redox-mediated electrochemical depolymerization of poly(dithiothreitol). (a) Scheme of the reaction. (b) Proposed mechanism.^54^

S–S bonds are principally found in vulcanized polymers in the tire industry. During chemical vulcanization, the double bonds in polyisoprene or poly(styrene–butadiene) react with sulfur to form cross-linked materials with several cyclic and mono-, di-, and polysulfide linkages.^51,52^ Nowadays in Europe nearly all used tires are recycled to recover energy or materials.^53^ However, one of the unsolved challenges in the chemical recycling of vulcanized polymers is to selectively cleave the C–S (273 kJ mol^−1^) and/or S–S (227 kJ mol^−1^) bonds over C–C bonds (347 kJ mol^−1^) for recovering of original polymers.^52^ The known methods [thermomechanical, thermochemical, physical (microwave, ultrasound) and biological] lead to partial oxidation, depolymerization or other degradation products which alter the properties of the devulcanized polymer. While electrochemical methods have not been reported yet on the devulcanization of polymer, a recent study from Daasbjerg *et al.* demonstrates a successful electrochemical-depolymerization of poly(dithiothreitol) derivatives *via* S–S bond cleavage using anthraquinone as a redox mediator (Fig. 5).^54^ These results could inspire further development of methodologies to selectively break C–S/S–S bonds to chemically recycle vulcanized polymers.

### 2.3. Electrochemical C–C bond cleavage

Polyolefins, including low-density polyethylene (LDPE), high-density polyethylene (HDPE), polypropylene (PP), polyvinylchloride (PVC) and polystyrene (PS), account for more than 60% of global plastic production and waste.^55^ They offer a wide range of properties (*e.g.*, lightweight, tunable mechanical strength, flexibility, chemical inertness) making them uniquely versatile materials. While the production of polyolefins is expected to quadruple by 2050, global recycling remains excessively low (5% for LDPE, 10% for HDPE, and <1% for PP).^5^ Current chemical methods for polyolefin recycling are mainly based on thermal degradation (*e.g.*, pyrolysis, gasification) that requires high temperatures, making the process energy-intensive.^56^ We refer the reader to the following reviews for a more detailed account of recent advances in the catalytic thermochemical recycling of polyolefins.^57–60^

**Fig. 6:**
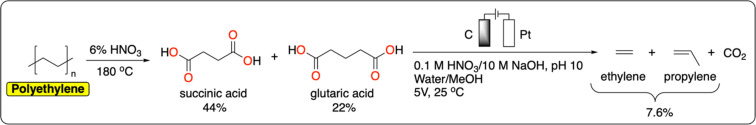
Integrated tandem chemical/electrochemical depolymerization of polyethylene to ethylene and propylene.^62^

The electrochemical cleavage of polar C–C or C=C bonds has been studied extensively on small molecules.^61^ However, there is still a large knowledge gap in approaches to breaking nonpolar C–C bonds such as those in polyolefins. Efficient electrochemical methods for the direct depolymerization of polyolefins back to monomers or building blocks have therefore not been reported to date. Nevertheless, recently Reisner *et al.* reported a tandem chemical-electrocatalytic process to recover a mixture of ethylene and propylene from polyethylene (Fig. 6). Both products were obtained from a Kolbe oxidation of succinic acid and glutaric acid generated from the chemical oxidation of polyethylene in 6% HNO_3_ at 180 °C.^62^ With this strategy, the authors managed to obtain a polyethylene-to-hydrocarbon (ethylene and propylene) yield of 7.6%. One of the main challenges in the further development of electrochemical oxidation methods to cleave nonpolar C–C bonds is to prevent overoxidation which ultimately leads to the decomposition of the polymer to CO_2_. For instance, Drogui *et al.* report an electrochemical method to decompose polystyrene microparticles to CO_2_. While this is not desirable from a recycling point of view, this approach has great potential to purify contaminated water streams containing micro and nanoparticles of plastics.^63^

### 2.4. Electrochemical C–N bond cleavage

**Fig. 7:**
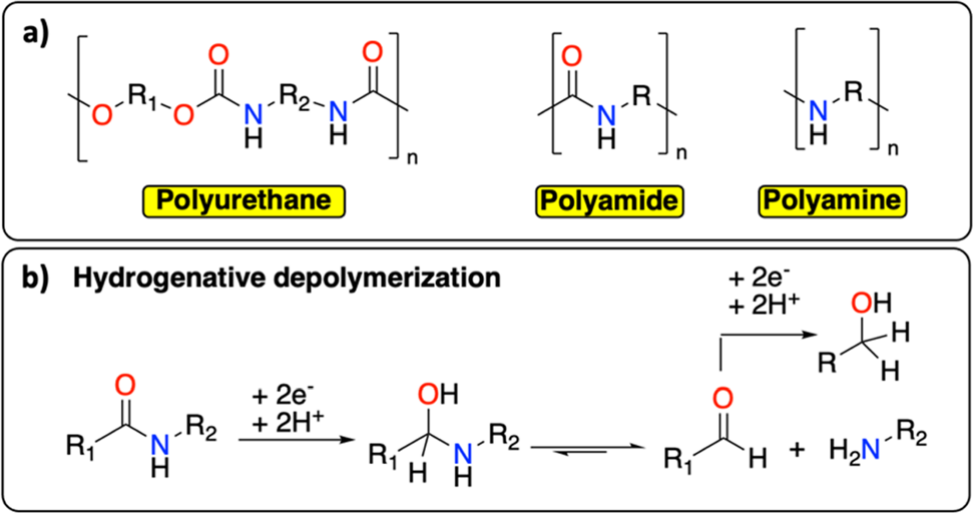
C–N containing polymers. (a) Schematic structure of polyurethane, polyamide and polyamine; (b) example of hydrogenative depolymerization of polyamide back to amine, aldehyde, or alcohol.

Following polyolefins, polymeric materials containing C–N groups constitute the second main class of polymers.^1^ This category includes polyurethanes, polyamides, and polyamines (Fig. 7a), which are extensively used as thermoplastic or thermosetting materials.^17^ They find a wide range of applications from rigid/flexible foams to coatings, fibers, adhesives, and elastomers.^64,65^ In Europe in 2017, the end-of-life management of this class of polymeric materials principally consisted of landfilling (0.9 Mt per year), incinerating (0.6 Mt per year) and recycling (mechanical and chemical, 0.5 Mt per year).^66^ The wide range of chemical structures, molecular weights, degrees of crystallinity, crosslinking densities, and hard-to-soft segment ratios make the chemical recycling of polymeric materials containing C–N groups particularly challenging.^65^ Similar to PET, the principal chemical recycling technique is solvolysis to (partially) recover monomers from polyurethanes and polyamides.^64,67^ For example, BASF, Maincoop and Polytecna, among others, have implemented this technique at the industrial scale to recover the polyol fractions from flexible polyurethane foams.^67^

Recently, Kumar and Milstein developed a versatile thermochemical hydrogenative depolymerization method where polyamides could partially be converted into the corresponding amino-alcohol with good conversion and selectivity.^68^ The proposed mechanism involves the hydrogenation of the amide bond leading to C–N bond dissociation and further hydrogenation of the formed aldehyde to the amino-alcohol (Fig. 7b). In the 1970s, Sabol *et al.* reported a similar reductive electrochemical approach on small aliphatic amides using an undivided electrochemical cell and two Pt electrodes.^69^ Through careful control of the reaction conditions, they could selectively isolate either the alcohol or the aldehyde product. We envision that a revisit of this methodology could open new doors for the (electro-)chemical recycling of C–N containing polymers.

## 3 Electrochemical post-polymerization functionalization

In post-polymerization functionalization (PPF), polymers undergo conversion to new polymers by editing the polymer backbone with new functional groups (Fig. 1c).^60^ Adjusting the degree of functionalization is a powerful means to fine-tune the thermal/mechanical/rheological properties of the resulting polymer without altering the bulk structure. These new polymers with tailored properties are especially attractive for specific/niche applications, that cannot or are difficult to be prepared through traditional “bottom-up” approaches. Alternatively, maintaining a low degree of functionalization enables the retention of the same properties as the parent polymers. Such an editing strategy is particularly appealing to introduce weak linkages into polymer backbones that are especially challenging to be chemically recycled such as polyolefins. PPF provides a way to add an end-of-life management tool into persistent polymers that have not been designed with this functionality.

While the first examples of electrochemical PPF methods have only recently been reported, thermochemical PPF methodologies have been developed for decades.^70^ These have been applied to a wide range of polymers such as polyesters, polyurethanes, polyethers and polyenes,^70–73^ as well as to the more challenging polyolefins.^60^ Most of these methods involve a change in the oxidation state of the polymer backbone. We therefore see great potential for electrochemical approaches, which could benefit from a more effective and controllable oxidation process (see Section 5). In this section, we summarize the main advances in electrochemical approaches for PPF. Similar to the first part, the discussions are organized by the type of chemical bond that is oxidized or reduced. When reported, we also briefly discuss the changes in the thermo/mechanical properties of the resulting polymer.

### 3.1. Electrochemical-oxidation of C–OH bonds

**Fig. 8:**
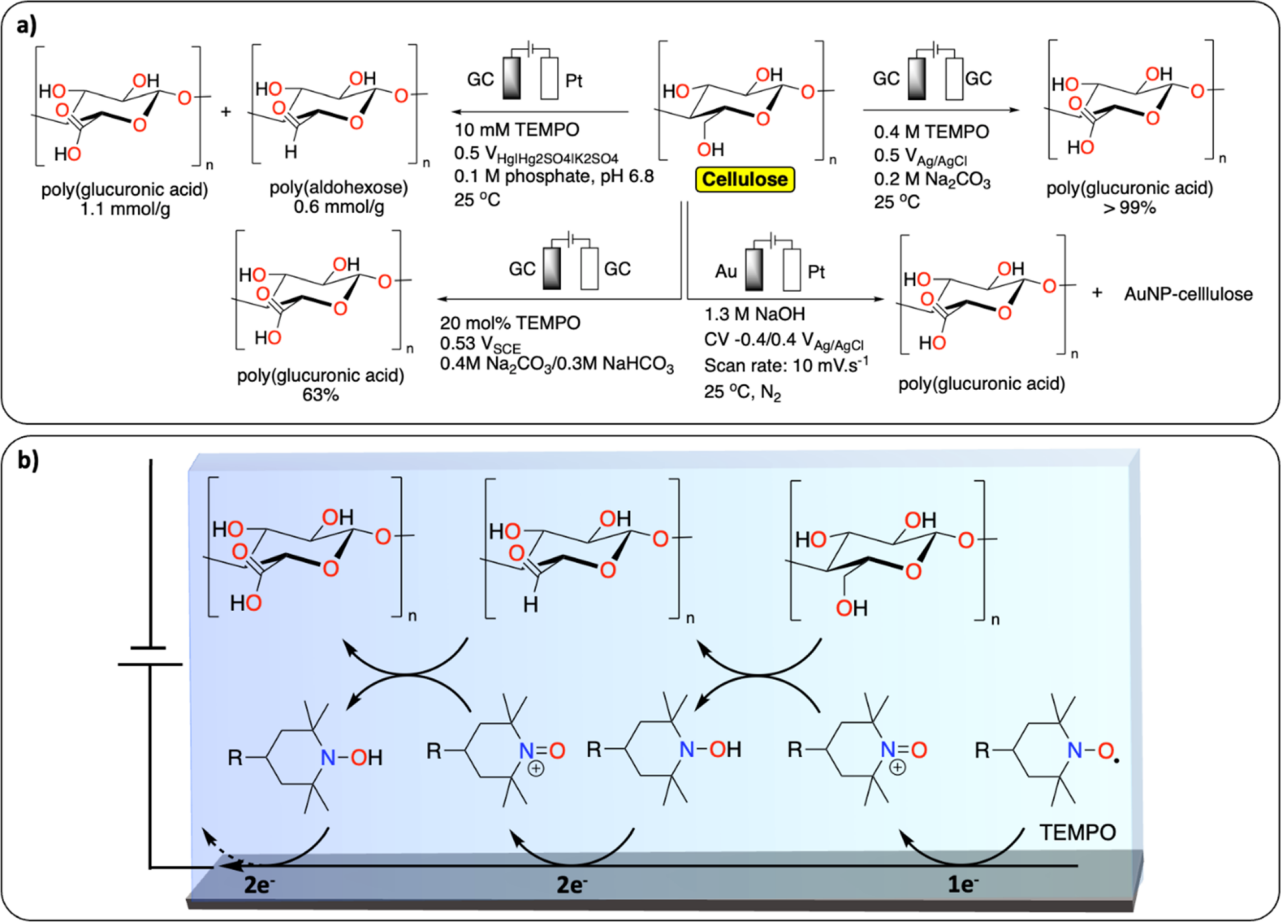
Electrochemical PPF of cellulose. (a) Scheme of reaction conditions reported for the electrochemical PPF of cellulose;^74–76,78^ (b) example of proposed mechanism of TEMPO mediated electrochemical oxidation of cellulose.

TEMPO (2,2,6,6-tetramethyl-1-piperidinyl-oxy) mediated oxidation of alcohol functionalities is the most reported strategy for the functionalization of cellulose (Fig. 8a).^74^ Isogai *et al.* reported on the TEMPO-mediated electrochemical oxidation of polysaccharides, including cellulose fiber. Interestingly, using this method the authors show that the cellulose fiber underwent little depolymerization – maintaining the original surface structure – while increasing aldehyde and carboxylic acid contents (Fig. 8b).^75^ A similar TEMPO-mediated oxidation of carbohydrates was reported to selectively oxidize the primary alcohols to carboxylic acids.^76^ More recently, Ivaska *et al.* investigated the mechanism of electrocatalytic oxidation of cellulose on gold electrodes.^77^ The main products were oxidized cellulose derivatives with increased carboxylic acid content and hybrid materials containing cellulose and gold nanoparticles. The group of Sugano further investigated the gold-catalyzed oxidation of hemicelluloses with similar results.^78^

### 3.2. Electrochemical oxidation of C–X bonds (X = Cl, F)

In Europe, approximately 27% (0.8 Mt) of waste PVC has been recycled and introduced back onto the market in 2022.^79^ Most of it has been mechanically recycled to generate materials with similar properties to pristine PVC. The challenge resides in properly sorting PVC waste to avoid cross-contamination of recycled PVC with the various toxic stabilizers (*e.g.*, cadmium, lead) and plasticizers (*e.g.*, phthalates) used to enhance the thermal stability or tune the properties of PVC for specific applications.^80,81^ Alternative recycling methods include the thermal and oxidative dechlorination of PVC to recover HCl, Cl_2_ and hydrocarbons.^81^

**Fig. 9:**
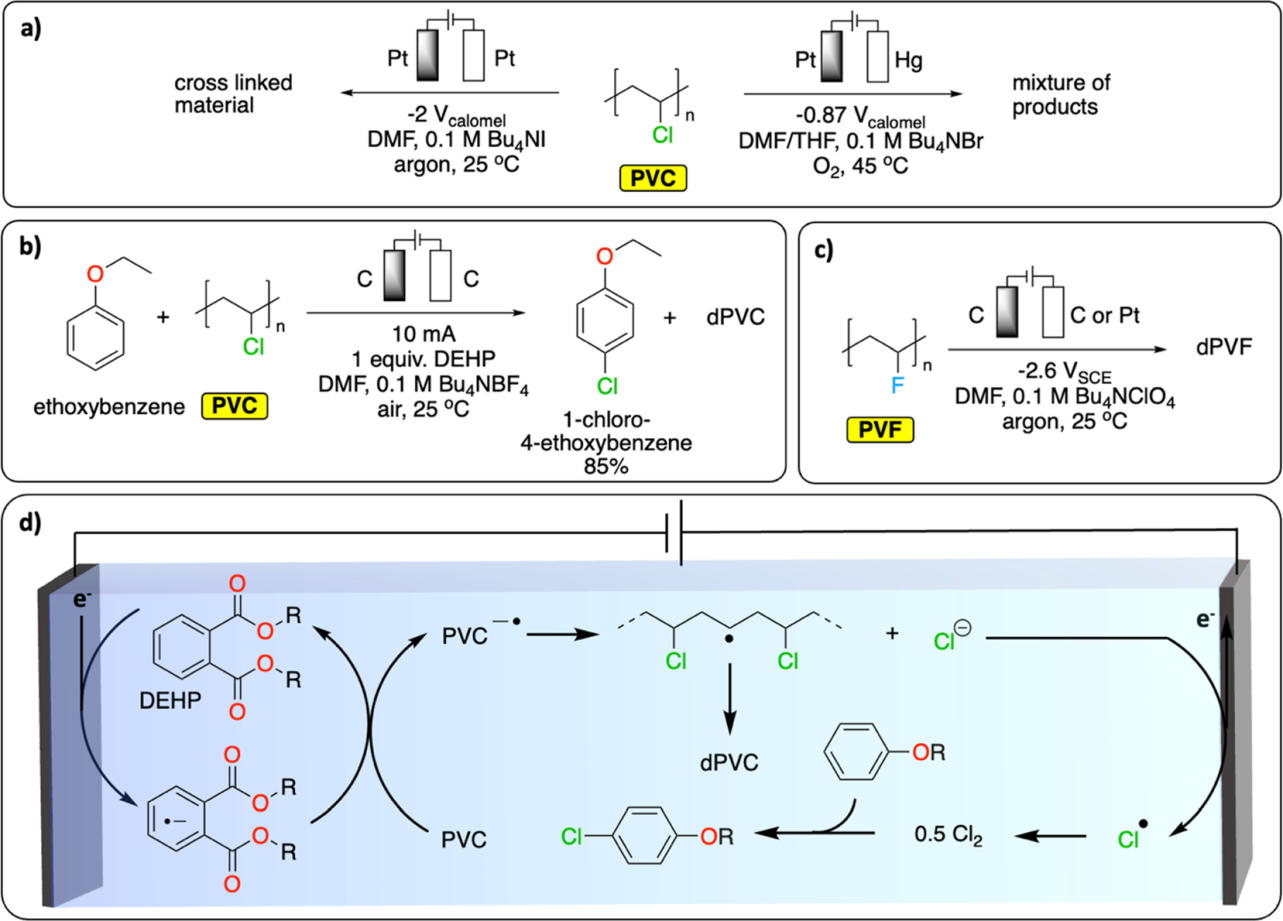
Electrochemical dehalogenation. (a) Electrochemical degradation of PVC; (b) tandem electrochemical dechlorination of PVC with concomitant chlorination of an electron-rich arene;^85^ (c) electrochemical defluorination of PVF;^89^ (d) schematic representation of the DEHP-mediated reductive dichlorination of PVC coupled with chlorination of an electron-rich arene. The abbreviations dPVC and dPVF refer to (partial) dechlorinated PVC and (partial) defluorinated PVF, respectively.^85^

Various electrochemical approaches have been developed for the reductive dehalogenation and oxidative halogenation of small molecules.^11,82,83^ On polymers, Batsalva *et al.* reported the reductive partial dehydrochlorination of PVC in DMF, on Pt electrode (Fig. 9a). They reported the formation of a cross-linked materials containing unsaturated groups that deposited on the cathode upon bulk electrolysis at −2 V *vs.* SCE.^84^ More recently, McNeil *et al.* reported the reductive partial dechlorination of PVC with concomitant oxidative chlorination of electron-rich arenes on graphite electrodes at room temperature in DMF electrolyte (Fig. 9b).^85^ Interestingly, di(2-ethylhexyl)phthalate, a common plasticizer additive used in PVC, served as a redox mediator to facilitate the reductive dechlorination reaction (Fig. 9d). While limited C–C bond scission was observed, the characterization of the resulting polymer reveals the presence of a complex mixture of functional groups including alkenyl, aromatic, ester/ether, alkyl and unreacted chloride groups. Consequently, the authors observed a substantial decrease in the glass transition temperature (*T*_g_, from 83 °C to 59 °C) and a higher degree of crystallinity in the resulting polymer. This proof of concept not only paves the way for paired-catalysis plastic recycling (the concept of paired-electrolysis is developed in Section 4) but also demonstrates that additives contained in plastics can even be beneficial for plastic electrochemical recycling. Besides reductive approaches, the oxidative dechlorination of PVC was initially studied by Ushida *et al.*, on Pt electrodes by electrogenerated superoxide anions, in THF/DMF mixed solvent (Fig. 9a).^86^ While dechlorination was achieved, substantial C–C bond cleavage was also observed together with the formation of hydroxy groups, conjugated carbonyl groups and polyene structures. In another study, the partial electrochemical degradation of PVC particles was investigated using hydrogen peroxide, which was generated from the oxygen reduction reaction on a TiO_2_/graphite cathode in an acidic aqueous solution at 100 °C. This method ultimately led to C–C bond cleavage, generating a mixture of dehalogenated polymers containing ketones and alcohol groups, oligomers and small molecules (formic acid, acetic acid and propionic acid).^87^

Similar to PVC, fluorinated polymers are mostly recycled mechanically.^88^ Chemical recycling principally concerns pyrolytic depolymerization to recover a mixture of fluorinated small molecules including tetrafluoroethylene. Apart from the issue of low selectivity, the process also requires high temperatures (>650 °C) and therefore a high energy input. Fluorinated polymers are well-known for their inertness to (electro-)chemical treatment. However, one report on polyvinyl fluoride (PVF) and polyvinylidene fluoride (PVDF) suggests that reductive defluorination is possible in DMF electrolyte on a glassy carbon electrode to form polyene-type materials (Fig. 9c).^89^

### 3.3. Electrochemical oxidation of C–H bonds

PPF of polyolefins provides an elegant alternative way of generating functionalized polyolefins, traditionally produced from the challenging olefin/polar monomer (*e.g.*, acrylates, amine-containing functional groups) copolymerization reaction.^90–92^ Over the years, various thermochemical approaches have been reported for C_sp^3^_–H functionalization of polyolefins (especially PE but also polyisobutylene, PP and PS), through oxidation, radical-based, carbene/nitrene, transition-metal catalyzed and dehydrogenation approaches.^60,73,93^

**Fig. 10:**
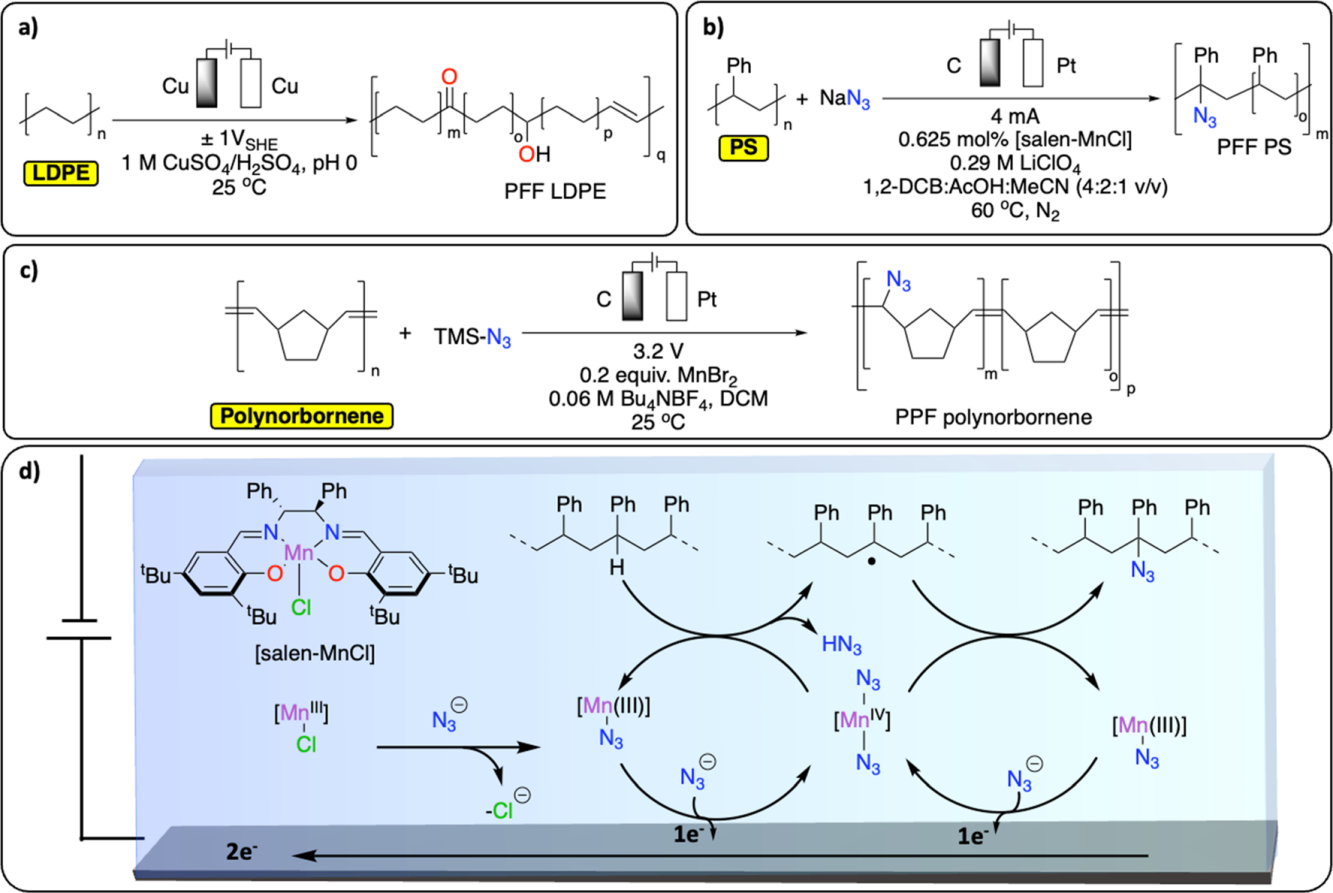
Electrochemical PPF strategies for C–H bond functionalization. (a) Electrochemical oxidation of LDPE;^96^ (b) electrochemical azidation of PS;^97^ (c) electrochemical azidation of polynorbornene;^98^ (d) schematic representation of the Mn-mediated oxidative azidation of PS.

Despite precedent on small molecules, reports on electrochemical methods for C_sp^3^_–H functionalization of polyolefins remain scarce.^94,95^ A recent study from the group of Botte demonstrates an electrochemical method to introduce ketones, alcohol functionalities and unsaturation in LDPE (Fig. 10a).^96^ By applying a low, oscillating cell potential of ±1 V *vs.* SHE on a Cu electrode, suspended LDPE in an acidic electrolyte containing CuSO_4_ was partially electrochemically oxidized, as observed by a significant increase of C=O, C–O and C=C signals in the post-catalysis IR spectrum of the resulting polymer.^97^ However, the approach also led to C_sp^3^_–C_sp^3^_ bond scission and the formation of smaller molecules such as dodecanoic acid.^96^ In another study, Ackermann *et al.* reported the electrochemical azidation of C_sp^3^_–H bonds in polystyrene (Fig. 10b).^97^ The C_sp^3^_–H azidation using a salen-type manganese complex afforded modified polystyrene materials with minimum C–C chain scission (up to 77% mass retention) and 1.6 mol% azidation using a graphite electrode. The high regioselectivity for the benzylic C_sp^3^_–H was achieved by careful design of the ligand motif to prevent side-reactions, including C–C bond breaking, Fig. 10d. The thermo/mechanical properties of the resulting azidated polymer show negligible changes compared to pristine PS. Electrochemical azidation reactions were also successfully performed on polynorbornene in an undivided cell (graphite anode/Zn cathode), in the presence of MnBr_2_ and trimethylsilyl azide in DCM (Fig. 10c). Interestingly, in a mixture of polynorbornene and PS, this method selectively cleaved C_sp^2^_–C_sp^2^_ bond of polynorbornene over PS.^98^

### 3.4. Electrochemical functionalization of aromatic groups

Polymers containing an aromatic ring in each repeating unit offer an additional handle for PPF. In particular, the introduction of functional groups onto PS, polysulfones, polyesters or polycarbonates is targeted to transform the polymers' properties for applications as compatibilizers, ion-exchange resins, or catalysts.^99^ In thermochemistry, these functionalization reactions are mostly performed *via* traditional electrophilic aromatic substitution (*e.g.*, Friedel–Crafts) although more exotic approaches have also been studied.^99^

**Fig. 11:**
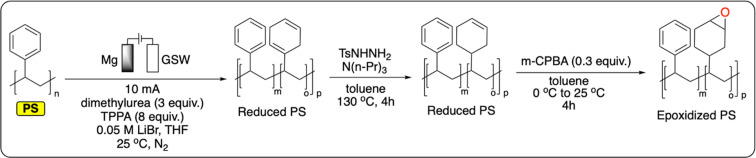
Electrochemical reduction of PS followed by chemical reduction and epoxidation.^100^

A recent study from Sarlah *et al.* demonstrates the electrochemical reductive dearomatization of polystyrene (Fig. 11).^100^ While the reaction was not possible using traditional Birch or Benkeser conditions, the authors adapted a previously reported method from Baran *et al.* on PS.^101^ A graphite cathode and a sacrificial Mg anode were used in a THF-based electrolyte to completely dearomatize commercial PS. Dimethylurea and hexamethylphosphoramide were used as additives to prevent the passivation of the cathode by lithium plating. Analysis of the resulting polymer showed no chain scission, with minor changes to the bulk thermoproperties of the material (*T*_g_ = 95–103 °C). Finally, it was demonstrated that the reduced polymer could serve as a platform for further functionalization by epoxidation of the formed cyclohexene rings (Fig. 11).^100^ This not only provides new functionalities for PS, it also gives a handle to tailor its *T*_g_ by controlling the degree of epoxidation (*T*_g_ = 128–162 °C). Next to this pioneering example, the functionalization of polymers containing aromatic groups has great potential for the electrochemical recycling of plastics. As previously discussed, electrochemical electrophilic aromatic substitution has been widely studied on small molecules. Once applied to aromatic polymers, this new strategy could be applied to prepare a wide variety of new materials.

## 4 Paired electrolysis

Paired electrolysis allows both half-reactions to generate valuable products in a complete electrochemical system, as opposed to the common focus on one of the half-reactions, where the other half-reaction only plays an auxiliary role. In an ideal process, all the electrons from the oxidized starting material at the anode (used as fuel) would be transferred to the reduced starting material at the cathode to achieve a combined electrochemical yield. The faradaic efficiency (FE) is then calculated by:
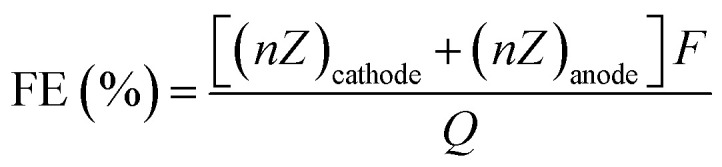
where *n*_cathode_ and *n*_anode_ are the moles of the desired products formed both at the cathode and the anode, respectively, *Q* is the charge passed, *Z*_cathode_ and *Z*_anode_ are the numbers of electron transferred to generate the product from starting materials at the cathode and anode, respectively, and *F* is the Faraday constant (96 485 C mol^−1^). In paired electrolysis, the FE can reach 200% in the case where all the electrons harvested at the anode are delivered at the cathode to selectively form the two desired products. BASF has developed the first commercial paired electrocatalysis process for the electrochemical oxidation of 4-*tert*-butylbenzene to 4-*tert*-butylbenzaldehyde with concomitant electrochemical reduction of phthalic acid dimethylester to phthalide with a FE as high as 180%.^102^

**Fig. 12:**
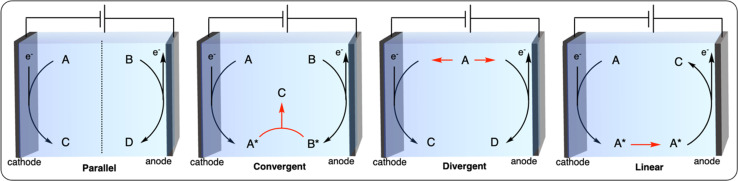
Schematic representation of the most common paired-electrolysis reactions.

Paired electrolysis can be categorized into four different approaches based on the interplay between two half-reactions: parallel, convergent, divergent, and linear (Fig. 12).^102^ In parallel paired electrolysis, distinct cathodic and anodic reactions occur simultaneously. In convergent paired electrolysis, the final products are formed through the reaction of intermediates generated from both the anode and cathode. In divergent paired electrolysis, the same substrate is converted to different products at the anode and cathode. Finally, in linear paired electrolysis, the substrate is converted to an intermediate at one electrode and converted to the final product at the other electrode. The vast possibilities of paired electrocatalysis open up new possibilities for the electrochemical recycling of plastics. Herein, we summarize the first examples of parallel and convergent paired electrolysis applied to plastic recycling. To the best of our knowledge, no plastic recycling strategies have been reported using the divergent and linear paired electrolysis approaches to date, leaving space for further development of novel dual catalysis methodologies.

**Fig. 13:**
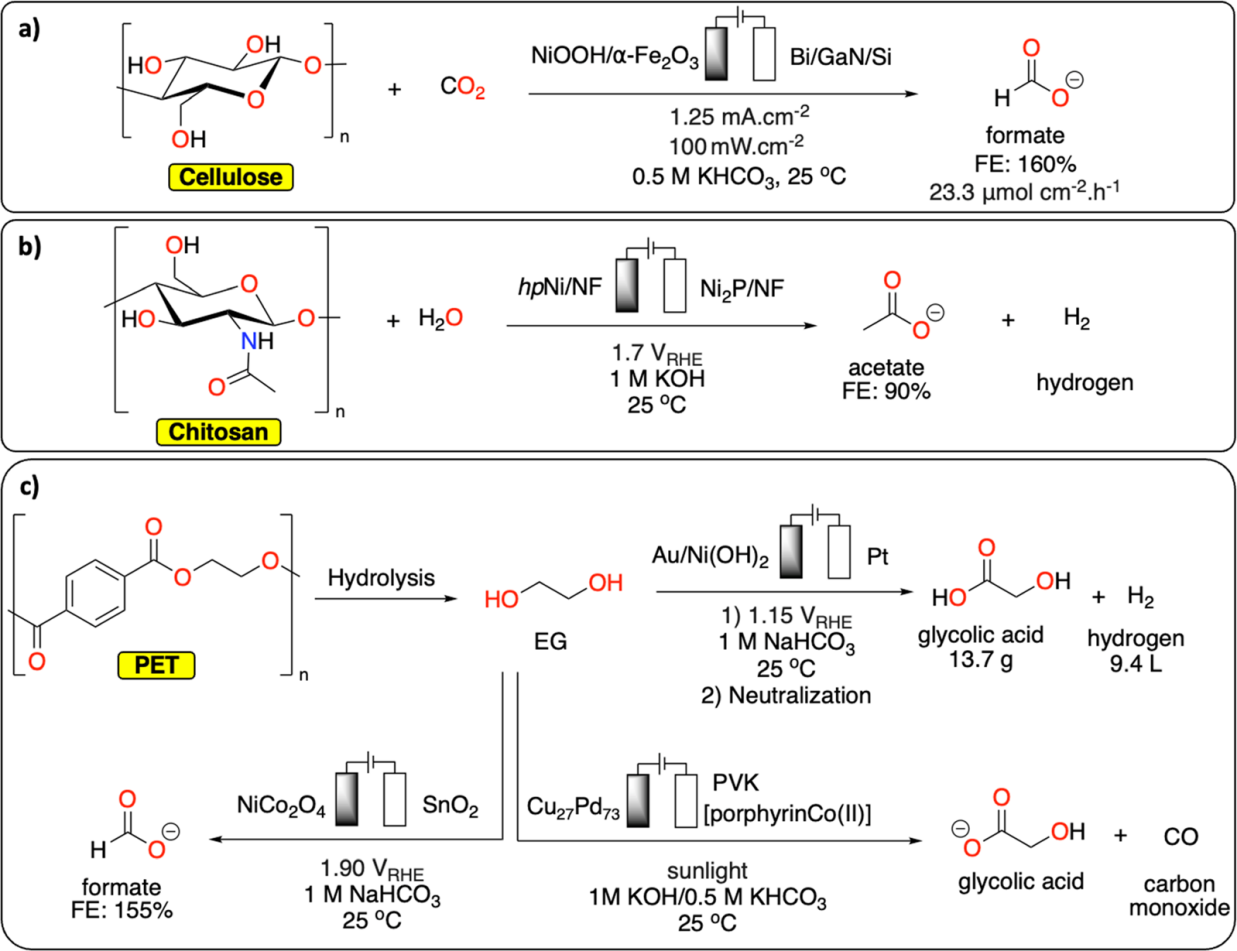
Parallel paired electrolysis polymer recycling methodologies. (a) Electrochemical conversion of cellulose/CO_2_ to formate;^106^ (b) electrochemical chitosan to acetate conversion with concomitant hydrogen production;^107^ (c) electrochemical conversion of EG/CO_2_ to various products.^16,108–110^

By carefully choosing the anodic and cathodic reactions, parallel paired electrolysis systems allow for the reduction of energy input of the electrochemical cell, in addition to the generation of two value-added products. In this context, the reduction of small molecules (*e.g.*, H_2_O, CO_2_) has been investigated in parallel paired electrolysis with oxidation of organic molecules derived from biomass (*e.g.*, HMF), as well as biopolymers.^103–105^ Chu *et al.* coupled the oxidation of a mixture of sugars derived from cellulose with CO_2_ reduction in a sunlight-driven electrochemical cell (Fig. 13a), producing formate at both electrodes in excellent faradaic efficiencies (>85%).^106^ By replacing conventional water oxidation at the anode with biomass conversion, the cell voltage was lowered by 32%. In a similar fashion, Li *et al.* reported on chitin oxidation to acetate with over 90% yield combined with hydrogen generation at the cathode, lowering the overall energy consumption with 15% compared to a system using conventional water oxidation (Fig. 13b).^107^

Although reports on direct polymer conversion with paired electrolysis are relatively scarce compared to other electrochemical topics, there are several promising examples using building blocks derived from polymer waste. For example, parallel paired electrocatalysis has successfully been used for EG recycling from PET waste. In a recent study from Ma *et al.*, EG produced from the alkaline depolymerization of PET was electrochemically oxidized to formic acid on a NiCo_2_O_4_ anode (Fig. 13c). The reaction was combined with the CO_2_ electrochemical reduction reaction on Sn electrode to also generate formic acid. Under optimized conditions, the parallel paired electrolysis generated formic acid with faradaic efficiency up to 155% at a cell voltage of 1.90 V.^108^ In another example from Duan *et al.*, EG from PET was electrochemically converted to glycolic acid on Au/Ni(OH)_2_ electrode with high selectivity (91%) and current density (326.2 mA cm^−2^) at 1.15 V *vs.* RHE while hydrogen was generated at the cathode (Fig. 13c).^16^ As a proof-of-concept, it was further demonstrated that by using a membrane-free flow electrolyzer, 13.7 g of glycolic acid and 9.4 L of H_2_ could be generated revealing the high potential of co-production of valuable chemicals and fuel from waste. Reisner *et al.* reported a photoelectrochemical approach to transform CO_2_ (from various feedstocks) into CO, syngas and formate while oxidizing EG from PET into glycolate using a perovskite-based photocathode containing an immobilized molecular Co-phthalocyanine catalyst and a Cu/Pd alloy anode (Fig. 13c).^109,110^ This study paves the way toward the development of integrated carbon capture and sunlight-driven utilization system for both CO_2_ and polymeric/plastic waste.

In convergent paired electrocatalysis, two different substrates undergo oxidation or reduction to afford intermediates/products that react among themselves to generate the final product. We previously described the work of McNeil *et al.* on the PPF of PVC which was paired with the chlorination of electron-rich arenes (Fig. 9d). This strategy could be seen as a convergent paired electrolysis where the HCl produced from the reduction of PVC reacts with the one-electron oxidized arene to form the final chlorinated arene. While this report constitutes the only example of convergent paired electrocatalysis applied to plastic recycling, it paves the way for future development (further discussed in Section 5) of using waste polymeric materials or plastics as reagents to form novel (polymeric) products.

## 5 Future directions and strategies

Polymer electrochemistry is an emerging field of electrochemistry providing complementary opportunities to traditional thermochemical approaches to notably tackle challenges in the recycling of polymeric materials. While considerable progress has been made in the electrochemical recycling of certain classes of polymers such as PET and biopolymers, a large gap in knowledge remains when it comes to the broader range of commodity and engineering polymeric materials and plastics. We therefore envision that the field will significantly grow in the coming years. Herein, we provide an outlook on future research directions in the field and highlight some of the most important challenges that need to be overcome.

### 5.1. Electrochemical activation of terminated polymer chains

**Fig. 14:**
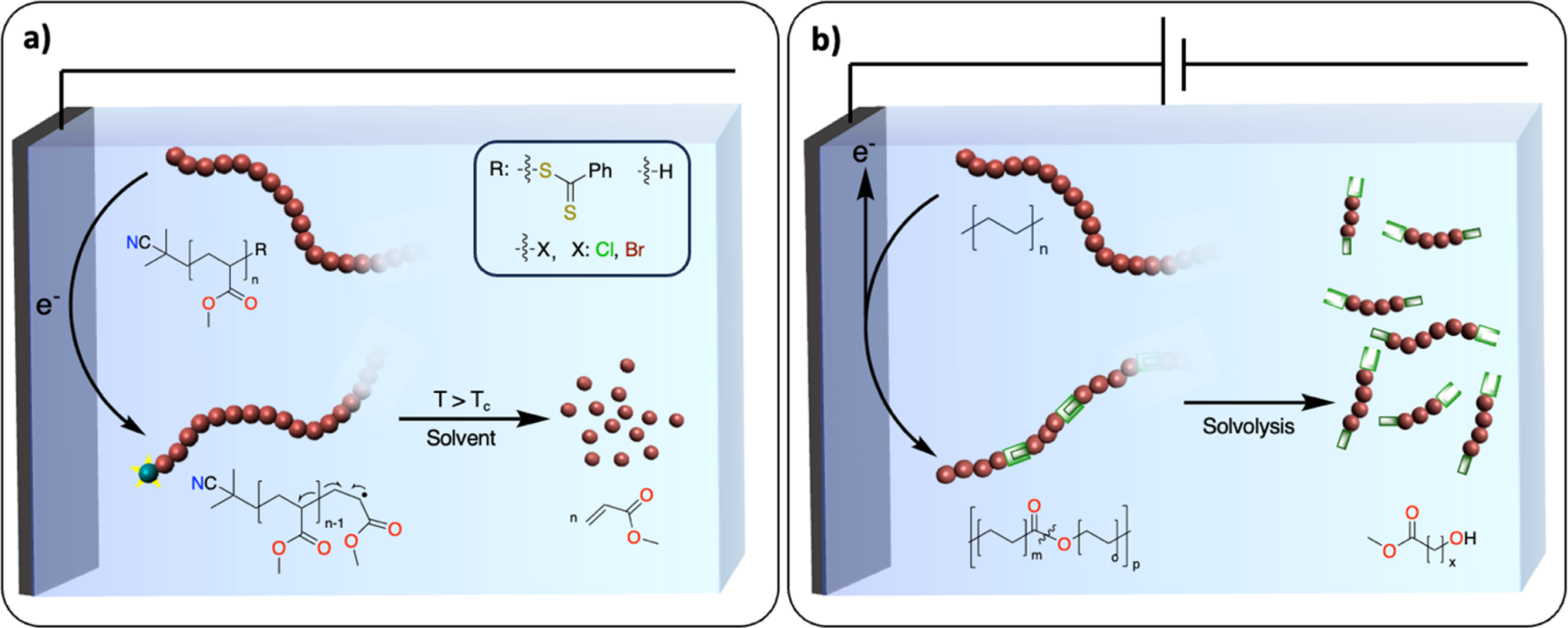
Two examples of future challenges for electrochemical recycling of polymeric materials. (a) Electrochemical activation of terminated polymer chains. The blue ball with the yellow star represents the activated chain-end of the polymer; (b) introducing weak linkages within the polymer backbone. The double green boxes represent the cleavable unit.

A polymerization reaction consists of three essential steps: initiation, propagation, and termination. Most exergonic polymerization reactions have a critical temperature (*T*_c_) at which the propagation stops. Above *T*_c_, depolymerization becomes thermodynamically favored and monomers can eventually be recovered from polymer chains that are not terminated. In plastic waste, polymer chains are terminated. One of the challenges is to find efficient methodologies to reactivate these dormant polymer chains, so that depolymerization may be possible at conditions above *T*_c_. In thermochemistry, this approach has been successfully developed for the depolymerization of commodity polymers synthesized *via* controlled radical (ATRP, RAFT, ITP), ionic, metathesis and ring opening-type polymerization.^111,112^ For instance, the group of Gramlich reported the radical depolymerization of Reversible Addition-Fragmentation Chain-Transfer RAFT-synthesized polymethylmethacrylate to recover methacrylate.^113^ Electrochemistry could provide a novel way to activate dormant polymer chains to induce depropagation and recover monomers with no concomitant waste formation. For instance, electrochemically mediated atom transfer radical polymerization has been studied extensively.^114^ It would be interesting to use similar strategies at conditions above *T*_c_ to recover monomers (Fig. 14a). It could also open new ways to recover monomers from polyolefins. A dual catalysis approach could be envisioned where electrochemically generated active polyolefin chains would be depolymerized *via* β-alkyl elimination using a thermocatalyst.^115^

### 5.2. Introducing weak linkages within the polymer backbone

With the inspiration from recent works on the redesign of polymers for circularity, the introduction of weak linkages within the polymer backbone represents an appealing strategy for closing the loop of the plastic economy.^116,117^ Taking the example of polyolefins, the polymer backbones solely consist of carbon–carbon repeating units, presenting no easily accessible handles for degradation/recycling. Recent work, pioneered by the group of Mecking, has demonstrated that synthesizing novel polymers consisting of isolated ester or ketone linkages embedded within an aliphatic C–C backbone affords recyclable/biodegradable polyolefin-like polymers.^117–119^ The insertion of weak linkages into backbones could also be used to convert polyolefin waste into recyclable counterparts while keeping the original properties. For instance, thermochemical oxidation of the polyolefin backbone enables the introduction of various functional groups such as alcohols, ketones, esters and oximes,^56,57,60,120^ which could serve as chemical handles for depolymerization reactions.^121^

The electrochemical oxidation of aliphatic C–H bonds on small molecules – notably on cyclohexane – to alcohols or ketones has been thoroughly investigated.^122^ There is also an example of successful electrochemically driven Baeyer–Villiger rearrangement to generate esters from ketones on cyclohexane-based substrates.^123^ In comparison with thermochemical approaches, electrocatalytic transformations favor reactions involving fewer proton and electron transfers, which may prevent the formation of overoxidized products, C–C bond scission and therefore CO_2_ release.^122^ Moreover, electrochemical oxidation generates active oxidants controllably and locally, which prevents the excess use of chemical oxidants needed in thermochemical reactions. Despite these promising studies, a selective method for the oxidation of polyolefins to polyesters (Fig. 14b) without substantial C–C bond breaking is still lacking.

### 5.3. Polymer-to-polymer electrochemical conversion

The polymer-to-polymer (electrochemical) conversion enables the synthesis of polymers with new properties from post-consumer plastics to target different types of applications. Using such a repurposing recycling strategy, plastic waste can be valorized by creating materials with higher economic value. For instance, waste polyethylene was successfully transformed into water-soluble antifungal polymers by thermochemical aerobic oxidation.^124^ In Section 3, we discussed the few reported examples of polymer-to-polymer electrochemical conversion demonstrating the potential of electrochemistry in performing such reactions. Herein, we want to go further into the possibilities that electrochemistry offers. Starting from PVC, the formation of a C-centered radical upon electrochemical dechlorination could be used as a platform to generate new polymers by reaction with various reagents (*e.g.*, CO_2_, Michael acceptors, alkynes, arenes), also with the possibility to use different paired electrolysis strategies. Similarly, the electrochemical functionalization of aromatic groups such as PS, PET, polycarbonate and polyurethane opens up new perspectives for the synthesis of novel polymers with totally different properties. However, it is important to mention that transforming a polymer into a new polymer implies that a novel strategy for its end-of-life management must be developed in parallel, otherwise, the approach merely delays the problem of waste plastic accumulation.

### 5.4. Challenges

In this section, we highlight some of the biggest challenges in the electrochemical recycling of polymeric materials and plastics to provide potential directions for future research. Although the discussion on these challenges may not be comprehensive, we hope to stimulate the community to develop new strategies and solutions to tackle these issues.

#### 5.4.1 Macromolecules *vs.* small molecules

Polymers are macromolecules, meaning they have completely different solution state properties compared to small molecules and are often insoluble in commonly used electrolytes. This requires adaptations in the way electrochemistry is typically done. For instance, in solution, high molecular weight polymers will slowly diffuse from/to active sites on the electrode surface for the electrochemical reaction to happen. This could lead to selectivity issues where part of the polymer is overoxidized/reduced while other parts remain unreacted. Similarly, for solid polymer particles, the electrochemical reaction could happen only on the surface, leaving the center of the particle untouched. In this case, side-reactions from the electrolyte such as the splitting of water into H_2_ and O_2_ would be favored, lowering the faradaic efficiency of the process. The development of novel concepts in the design of (homogeneous and heterogeneous) electrocatalysts and redox mediators is therefore essential. Examples of this would be electrodes with large pores which polymers could effectively diffuse into and redox mediators which could diffuse into insoluble plastic particles.^125,126^ The development of novel electrolytes and the engineering of suitable electrochemical cells is equally essential. In the field of lignin valorization, the wide variety of products formed during its electrochemical oxidation has led to the development of an array of approaches to deal with selectivity issues that could inspire the plastic electrochemical recycling community. Firstly, the use of ionic liquid-based electrolytes has demonstrated a significant improvement in controlling the selectivity of reactions due to a larger solvent window and a better solubility of lignin.^127,128^ Secondly, careful selection of catalysts and additives significantly improves the selectivity of electrochemical reactions.^50^ Thirdly, to avoid the overoxidation of lignin at the anode, advanced extraction methods have been developed, such as the use of an anionic exchange resin to absorb products,^129^ or the constant separation of the products from the electrolyte using a nanofiltration membrane.^130^

#### 5.4.2 Impurities in plastic waste streams

In most current electrochemical systems, relatively pure feeds, electrolytes, and solvents are needed to obtain the best performances. Impurities can impact the outcome of these reactions, especially by altering electrocatalysts over time. When working with real-world waste plastics, the presence of additives, contaminants (metallic and organic), plasticizers, pigments, and other non-polymeric components must be considered.^131^ Although some additives may be beneficial, such as in McNeil's example of electrochemical PVC recycling with phthalate additives,^85^ most often it is more likely that they will significantly damage the electrolyzer. This damage may occur through the deposition of organic or metallic impurities on the working electrode, the deterioration of the membrane separating the cathode from the anode or the presence of side reactions, lowering the faradaic efficiency of the reaction. While new technologies must be developed to minimize the concentration of contaminants, future research should consider the development of robust electrolyzers tolerant to such impurities. For instance, it could involve the use of strong chelating ligands (*e.g.*, EDTA, TMEDA)^132^ – typically used in conjunction with sacrificial electrodes – as additive in the electrolyte to prevent the electroplating of metal impurities. The recent development of hybrid electrodes,^133^ consisting of an electrocatalyst coated with a film could provide additional solutions where only polymers could diffuse to the active sites of the electrode while small organic molecules could not.

#### 5.4.3 Products isolation and energy penalty

Isolation of products soluble in electrolytes is a common concern in electrochemistry.^134,135^ The purification step is particularly energy intensive with products that are formed in low concentration (nM to μM) and sometimes in exotic electrolyte. While this is not an issue for fundamental research and proof-of-concept studies, it becomes an issue for potential applications. One way to reduce this energy penalty is to increase product concentration in the electrolyte. However, this comes with other challenges such as change in the selectivity profile of the catalyst and formation of byproducts by over-oxidation or reduction. It is therefore important to study the effects of product accumulation on the performance of the electrolyzer. Other approaches involve systematic studies and reaction optimization in various electrolytes but also new technological solutions such as advanced extraction methods as discussed in Section 5.4.1 and novel electrochemical reactor designs (*e.g.*, new flow reactors adapted to insoluble polymeric materials).^136^

#### 5.4.4 Benchmarking

In the emerging field of electrochemical recycling of polymeric materials, establishing benchmark criteria is crucial.^137^ In typical electrocatalysis with small, well-defined molecules, substrate and intermediate absorption can be directly observed through spectroscopy, followed by the detection of products. This allows for a clear assessment of catalytic properties and the identification of crucial factors for optimization. In contrast, the interaction between heterogeneous macromolecules and electrodes, as well as the reaction route, remains unclear. This poses challenges for optimizing and innovating electrochemical recycling systems for polymeric material waste. Furthermore, the variation in crucial polymer parameters, such as molecular weights, dispersity, solubility, and degree of crystallinity (if any), necessitates exploration into how these factors influence electrochemical recycling. The impact of the polymer properties on electrocatalysts, including size, porosity, and density of particles/films, requires thorough exploration and is often overlooked in current studies. Establishing benchmarks is essential for ensuring reproducibility across the community and avoiding non-comparable isolated cases.

#### 5.4.5 Characterizations of post-polymerization functionalized polymers

Proper characterization of PFF polymers offers insights into the structure/property relationships but also into the mechanisms of reactions. With current analytic methods, it is difficult to know if PPF occurs on all polymer chains but also if it is homogeneously spread or localized on one part of a polymer chain. This is a recurrent issue in the broader field of polymer chemistry which has received insufficient attention. While indirect studies on small analogues provide valuable insights, disparities with real macromolecular counterparts should not be overlooked (*cf.* macromolecules *vs.* small molecules). In light of these considerations, we briefly discussed some limitations of current characterization tools as well as potential solutions to overcome these challenges.

Firstly, conventional NMR spectroscopy techniques employed for solution state material characterization often fail to accurately map and depict the degree of post-polymerization functionalization, particularly in case of a low degree of functionalization. Isotopic labelling such as ^13^C, ^18^O, ^15^N, and/or fluor/phosphor probes offers additional avenues for characterization but may pose challenges to introduce within the polymer backbone or require the use of costly chemicals.^129,130^ Less conventional NMR spectroscopy techniques such as solid state and Dynamic Nuclear Polymerization (DNP) NMR spectroscopy which are increasingly recognized as powerful and versatile tools for the characterization of polymeric materials, are still underutilized in the field but could provide valuable complementary insights to traditional solution state NMR spectroscopy techniques.^138,139^

Secondly, optical microscopy can offer essential information about the degree of functionalization of modified polymers, although this method requires additional fluorescent decoration.^131^ For instance, the groups of Chen and Coates have reported a powerful tool to monitor the precise monomer sequencing at a single polymer chain level on various synthetic polymers using a coupled reaction approach toward super-resolution imaging.^140^ Developing a reminiscent versatile method to characterize PPF polymers would be of high interest to this field of research.

Thirdly, the characterization of polymers on GPC/SEC with multiple detectors is a routine technique in polymer chemistry to extract a plethora information on polymers (*e.g.*, *M*_n_, *M*_w_, PDI, viscosity, branching, cross-linking). Specifically, GPC/SEC-IR is a powerful tool to analyze the degree of functionalization as a function of elution time. However, the development of novel detectors for a quantitative measure when the degree of functionalization is below 5%, range typically achieved in most PPF on synthetic polymers, is needed.

Finally, atomic force microscope coupled with IR (AFM-IR) provides a means to characterize both physical properties (*e.g.*, surface stiffness, morphology) and chemical compositions of polymers, offering useful information on the degree and spatial distribution of functionalization on 2D polymeric films.^132^

## Author contributions

All authors wrote, revised, and edited the manuscript.

## Conflicts of interest

The authors declare no conflict of interest.

## Supplementary Material
